# Macrolide-Resistant *Bordetella pertussis*, Vietnam, 2016−2017

**DOI:** 10.3201/eid2610.201035

**Published:** 2020-10

**Authors:** Kazunari Kamachi, Hong T. Duong, Anh D. Dang, T. Hai, Do Do, Kentaro Koide, Nao Otsuka, Keigo Shibayama, Ha Thi Thu Hoang

**Affiliations:** National Institute of Infectious Diseases, Tokyo, Japan (K. Kamachi, K. Koide, N. Otsuka, K. Shibayama);; National Institute of Hygiene and Epidemiology, Hanoi, Vietnam (H.T. Duong, A.D. Dang, H.T.T. Hoang);; Center for Pediatric Tropical Diseases, National Hospital of Pediatrics, Hanoi (H.T. Do)

**Keywords:** Bordetella pertussis, macrolide resistance, 23S rRNA, Vietnam, A2047G mutation, genotyping, respiratory infections, bacteria, antimicrobial resistance

## Abstract

Macrolide-resistant *Bordetella pertussis* emerged in Vietnam during 2016−2017. Direct analyses of swab samples from 10 patients with pertussis revealed a macrolide-resistant mutation, A2047G, in the 23S rRNA. We identified the MT104 genotype of macrolide-resistant *B. pertussis* (which is prevalent in mainland China) and its variants in these patients.

Pertussis (whooping cough) is a highly contagious disease caused by the gram-negative bacterium *Bordetella pertussis*. Vaccination is an effective method to prevent and control pertussis, but in many countries, pertussis incidence remains despite high vaccination coverage. Macrolides are commonly used to treat pertussis, but macrolide-resistant *B. pertussis* (MRBP) strains have been observed in mainland China and Iran (*1*–*4*). In China, MRBP is isolated with increasing frequency (57.5%−91.9%) and has been since the early 2010s (*4*,*5*). Most MRBP isolates from China have a homogeneous A2047G mutation in each of the 3 copies of the 23S rRNA gene, which is associated with macrolide resistance (*1*,*3*,*4*). In contrast, MRBP is rare in Iran; the A2047G mutation is not identified in the Iran MRBP isolate (*6*). China has several reports of MRBP, but our knowledge about these bacteria in other countries in Asia is limited.

To survey MRBP in Vietnam, which neighbors China, we performed a retrospective analysis of stored DNA samples from nasopharyngeal swabs collected during 2016–2018 from 53 patients with pertussis in northern Vietnam (median age 3 months [range 31 days–32 years]; 14 patients in 2016, 38 in 2017, and 1 in 2018) (Appendix Table). Nucleic acid amplification testing was used to diagnose *B. pertussis* in patients who were infected with this condition. We used the cycleave real-time PCR targeting the A2047 mutation in the *B. pertussis* 23S rRNA to examine the 53 DNA samples (Appendix). Of these DNA samples, 10 (19%) were positive for the A2047G mutation. PCR-based sequencing validated the presence of the mutation (*3*). Nine of these samples were from infants 32 days–4 months of age, and 1 was from a woman 29 years of age (Table). Geographically, 7 DNA samples were found in Hanoi and 3 in other provinces (Ha Nam and Thai Binh) (Appendix Figure 1). Five patients were treated with β-lactam antimicrobial drugs; the treatments for other patients and their epidemiologic links are unknown.

**Table:** Direct genotyping of *Bordetella pertussis* with the detected macrolide-resistant A2047G mutation in the 23S rRNA gene, Vietnam, 2016−2017*

Patient no.	Age/sex	Year/province	MLVA type	Repeat no. VNTRs†	Allele type of virulence-associated genes‡				C5330 in *fhaB*§
					*ptxP*	*ptxA*	*prn*	*fim3*	
1	2.5 mo/M	2016/Hanoi	MT104	8/6/0/7/6/10	1	1	1	A	NA
2	2 mo/F	2016/Ha Nam	New type A	8/6/0/6/6/10	1	1	1	A	C5330T
3	32 d/F	2016/Hanoi	New type B	9/6/0/7/6/10	1	1	1	A	C5330T
4	3 mo/F	2016/Hanoi	MT104	8/6/0/7/6/10	1	1	1	A	C5330T
5	2 mo/M	2016/Hanoi	MT104	8/6/0/7/6/10	1	1	1	A	C5330T
6	29 y/F	2016/Hanoi	MT104	8/6/0/7/6/10	1	1	1	B	C5330T
7	4 mo/F	2017/Thai Binh	MT104	8/6/0/7/6/10	NA	1	1	B	C5330T
8	52 d/F	2017/Ha Nam	MT104	8/6/0/7/6/10	1	1	1	A	C5330T
9	3 mo/M	2017/Hanoi	MT104	8/6/0/7/6/10	1	1	1	A	C5330T
10	3 mo/M	2017/Hanoi	MT104	8/6/0/7/6/10	1	1	1	A	C5330T

*MLVA, multilocus variable-number tandem-repeat analysis; NA, not analyzed; VNTR, variable-number tandem-repeat. †The order is VNTR1/VNTR3a/VNTR3b/VNTR4/VNTR5/VNTR6. ‡*B. pertussis* virulence–associated allelic genes (*ptxP*, *ptxA*, *prn*, and *fim3*). *§fhaB3* allele carries the single-nucleotide polymorphism mutation C5330T.

We used multilocus variable-number tandem-repeat analysis (MLVA) to determine the genotypes of the MRBP by direct genotyping (*7*). We classified the MLVA profiles into the following 3 genotypes: MT104 (n = 8) and new genotypes A and B (n = 1 each) (Table). Genotypes A and B were minor single-locus variants of MT104, differing in 1 of the 6 variable-number tandem-repeat (VNTR) loci. Phylogenetic analysis revealed that the MRBP belonging to genotypes A and B were closely related to MT104 (Appendix Figure 2). We also characterized *B. pertussis* virulence–associated allelic genes (*ptxP*, *ptxA*, *prn*, and *fim3*) by DNA sequence-based typing (*7*). Of the 10 MRBP DNA samples, 9 yielded a complete profile of virulence-associated allelic genes, 8 were *ptxP1/ptxA1/prn1/fim3A*, and 1 was *ptxP1/ptxA1/prn1/fim3B* (Table). The allelic profile *ptxP1/ptxA1/prn1* is common in MRBP strains prevalent in China (*8*). In addition, 9 of the MRBP DNA samples exhibited the C5330T mutation in *fhaB3*, which is frequently observed in MRBP in China (*9*) (Appendix Table).

Genotyping assays revealed that MRBP strains in Vietnam were closely related to an MRBP strain identified in China. The major MLVA types reported recently in China are MT55, MT104, and MT195 (*8*,*9*). These types are closely related; they have only 1 difference at 1 VNTR locus. All isolates of these genotypes contained the macrolide resistance A2047G mutation in the 23S rRNA. A clinical strain of MT104-MRBP was first identified in 2012 in Shannxi, China. Subsequently, this clinical strain of MT104-MRBP was found throughout the country (*9*).

In Vietnam, the *B. pertussis* population comprises 2 major strains, MT27 and MT104 (Appendix Figure 2). The MT27 strain is common in industrialized countries but not in China (*8*,*9*). In contrast, the MT104 strain is not common in industrialized countries but frequent in China. We define a clonal complex as genotypes differing in only 1 of the 6 VNTRs. We have 2 clonal complexes in the *B. pertussis* population in Vietnam, 1 containing MT104 and genotypes A and B and another containing MT18, MT27, and MT28. MRBP genotypes A and B differ from MT104 by a single repeat at 1 VNTR locus. MRBP genotypes A and B are grouped within the clonal complex of MRBP. This finding suggests that the MRBP-MT104 strain was imported from China to Vietnam before 2016 and subsequently mutated to genotypes A and B over time. Macrolides are the third most common antimicrobial drugs used in Vietnam (*10*), and they are commonly available at private pharmacies without prescriptions, suggesting that the uncontrolled use of macrolides might have selected MRBP in the country.

In conclusion, we reported the emergence of MRBP in Vietnam during 2016−2017. We detected MRBP strains that have the same or a similar phylogenetic lineage as 1 of the MRBP strains prevalent in China. Because MRBP is a serious threat to public health, global surveillance of MRBP is needed, especially in countries in Asia.

Appendix Additional information about a retrospective analysis of stored DNA samples from nasopharyngeal swabs collected during 2016−2018 from 53 patients with pertussis, northern Vietnam.
