# Supplementary material for: Macrolide-Resistant Bordetella pertussis, Vietnam, 2016−2017
Source: Emerg Infect Dis. 2020 Oct;26(10):2511–3. doi: 10.3201/eid2610.201035 (PMC7510698; doi:10.3201/eid2610.201035)
Supplement: Appendix — Additional information about a retrospective analysis of stored DNA samples from nasopharyngeal swabs collected during 2016−2018 from 53 patients with pertussis, northern Vietnam. [file 20-1035-Techapp-s1.pdf]

Article DOI: https://doi.org/10.3201/eid2610.201035

Macrolide-Resistant *Bordetella pertussis* in Vietnam, 2016–2017

Appendix

Cycleave Real-Time PCR Assay for Detection of the A2047G Mutation

A cycling probe technology assay was used (*1*,*2*). To discriminate between the mutant allele A2047G and wild-type allele (non-A2047G) of 23S rRNA in *B. pertussis*, two cycling probes were designed: 5′-Eclipse-GACGGgAAG-HEX-3′ for A2047G and 5′-Eclipse-AGACGGaAAG-FAM-3′ for non-A2047G. The upper- and lowercase letters in the sequences indicate DNA and RNA, respectively. The duplex Cycleave real-time PCR was performed using an ABI 7500 Fast Real-Time PCR System (Applied Biosystems). PCR amplification was carried out in 20 µL reactions containing 10 µL of 2× CycleavePCR Reaction Mix (Takara Bio Inc, Japan), 0.4 µL of 50× ROX reference dye II (Takara Bio), 2 µL of DNA sample, 0.2 µM of each cycling probe, and 0.2 µM of each primer (Primer-F: 5′-GAATGGCGTAACGATG-3′ and Primer-R: 5′-TGCAAAGCTACAGTAAAGG-3′). The PCR conditions were the following: 20 s at 95°C, followed by 40 cycles of 95°C for 3 s, 60°C for 10 s, and 72°C for 25 s. The fluorescence signal of HEX was monitored on the VIC channel of the PCR system. In each assay, two PCR fragments (A2047- and A2047G-DNA fragments, 5× 10^3 DNA copies/µL each) were used as positive controls; sterile distilled water was used as a negative control. The detection limits of the PCR assay were »20 DNA copies/tube for both positive controls.

Construction of A2047- and A2047G-DNA Fragments

The positive controls (796 bp each) were constructed by PCR using *B. pertussis* Tohama DNA as a template (non-A2047G strain). The A2047-DNA fragment (wild-type allele) was amplified using the primers 23S rDNA-F (5′-GGTATACCCTGGTAGTGTGAAG-3′) and 23S rDNA-R (5′-CGACATCGAGGTGCCAAA-3′), and purified with the MinElute PCR Purification Kit (Qiagen). The A2047G-DNA fragment (mutant allele) was constructed by

overlap extension PCR method (*3*). Briefly, two DNA fragments were amplified by PCR using the primer sets, 23S rDNA-F and 23S A2047G-R (5′-AAGGTTCATGGGGTCTTCCCGTCTAGCCGCGGGTA-3′), and 23S A2047G-F (5′-TACCCGCGGCTAGACGGGAAGACCCCATGAACCTT-3′) and 23S rDNA-R, respectively. The DNA fragments were joined by overlap extension PCR using the primers 23S rDNA-F and 23S rDNA-R and purified with MinElute PCR Purification Kit. The substitution of A with G (at position 2047) in the primer sequences has been underlined.

References

1. Duck P, Alvarado-Urbina G, Burdick B, Collier B. Probe amplifier system based on chimeric cycling oligonucleotides. Biotechniques. 1990;9:142–8. PubMed

2. Bekkaoui F, Poisson I, Crosby W, Cloney L, Duck P. Cycling probe technology with RNase H attached to an oligonucleotide. Biotechniques. 1996;20:240–8. PubMed https://doi.org/10.2144/96202rr01

3. Ho SN, Hunt HD, Horton RM, Pullen JK, Pease LR. Site-directed mutagenesis by overlap extension using the polymerase chain reaction. Gene. 1989;77:51–9. PubMed https://doi.org/10.1016/0378-1119(89)90358-2

Appendix Table. Analysis of *Bordetella pertussis* in the DNA samples collected from 53 pertussis patients, Vietnam, 2016–2018*

Year of specimen collection	Province	Age/sex	Vaccine status	A2047G in 23S rRNA	MLVA type	Allele type of virulence-associated genes†				C5330 in *fhaB*‡
						ptxP	*ptxA*	*prn*	*fim3*	
2016	Hanoi	32 y/F	3 doses	Negative	MT18	3	1	2	A	C
2016	Hanoi	5 m/F	2 doses	Negative	MT27	3	1	2	A	C
2016	Hanoi	28 y/F	3 doses	Negative	MT27	3	1	NA	B	NA
2016	Cao Bang	26 y/F	Unknown	Negative	MT27	3	1	2	A	C
2016	Phu Tho	4 m/F	1 dose	Negative	NA	ND	ND	ND	ND	ND
2016	Phu Tho	3 m/F	1 dose	Negative	MT27	3	1	2	A	NA
2016	Hanoi	2.5 m/M	Unknown	Positive	MT104	1	1	1	A	NA
2016	Bac Giang	2 m/M	0	Negative	MT27	3	1	2	B	NA
2016	Ha Nam	2 m/F	0	Positive	Novel type A	1	1	1	A	C>T
2016	Hanoi	32 d/F	0	Positive	Novel type B	1	1	1	A	C>T
2016	Hanoi	3 m/F	1 dose	Positive	MT104	1	1	1	A	C>T
2016	Hanoi	2 m/F	0	Negative	NA	ND	ND	ND	ND	ND
2016	Hanoi	2 m/M	0	Positive	MT104	1	1	1	A	C>T
2016	Hanoi	29 y/F	3 doses	Positive	MT104	1	1	1	B	C>T
2017	Thai Binh	4 m/F	2 doses	Positive	MT104	NA	1	1	B	C>T
2017	Hanoi	2 m/F	0	Negative	NA	ND	ND	ND	ND	ND
2017	Cao Bang	11 m/M	Unknown	Negative	NA	ND	ND	ND	ND	ND
2017	Cao Bang	3 y/M	3 doses	Negative	Novel type C	1	1	1	A	C
2017	Cao Bang	4 y/F	3 doses	Negative	NA	ND	ND	ND	ND	ND
2017	Cao Bang	2 m/F	0	Negative	Novel type C	1	1	1	A	C
2017	Hanoi	2 y/M	3 doses	Negative	MT27	3	1	2	A	C
2017	Vinh Phuc	8 y/F	3 doses	Negative	MT27	3	1	2	A	C
2017	Vinh Phuc	2 m/M	0	Negative	MT27	3	1	2	A	C
2017	Hai Duong	4 m/F	2 doses	Negative	MT28	3	1	NA	B	C
2017	Thai Binh	4 m/M	1 dose	Negative	NA	ND	ND	ND	ND	ND
2017	Thai Binh	3 m/M	1 dose	Negative	MT27	3	1	2	A	C
2017	Hai Duong	45 d/M	0	Negative	MT28	3	1	2	A	NA
2017	Hanoi	3 m/F	1 dose	Negative	NA	ND	ND	ND	ND	ND
2017	Thai Binh	31 d/M	0	Negative	Novel type D	3	1	2	A	NA
2017	Hai Duong	52 d/M	0	Negative	NA	ND	ND	ND	ND	ND
2017	Cao Bang	6 y/M	3 doses	Negative	NA	ND	ND	ND	ND	ND
2017	Ha Giang	5 m/M	Unknown	Negative	NA	ND	ND	ND	ND	ND
2017	Ha Giang	18 m/F	3 doses	Negative	NA	ND	ND	ND	ND	ND
2017	Ha Tinh	2 m/M	Unknown	Negative	NA	ND	ND	ND	ND	ND
2017	Hanoi	20 m/F	3 doses	Negative	NA	ND	ND	ND	ND	ND
2017	Ninh Binh	4 m/F	1 dose	Negative	MT27	3	1	2	A	C
2017	Cao Bang	4.5 y/M	3 doses	Negative	MT27	3	1	NA	NA	NA
2017	Hai Duong	2 m/M	0	Negative	NA	ND	ND	ND	ND	ND
2017	Hai Duong	2 m/M	0	Negative	Novel type C	1	1	1	A	C
2017	Hai Duong	4 m/M	2 doses	Negative	MT27	3	1	2	A	C
2017	Hanoi	2 m/F	0	Negative	MT27	3	1	NA	B	C
2017	Hanoi	3 m/F	1 dose	Negative	NA	ND	ND	ND	ND	ND
2017	Ha Nam	52 d/F	0	Positive	MT104	1	1	1	A	C>T
2017	Tuyen Quang	45 d/F	Unknown	Negative	NA	ND	ND	ND	ND	ND
2017	Hanoi	3 m/M	1 dose	Positive	MT104	1	1	1	A	C>T
2017	Hanoi	23 y/F	3 doses	Negative	MT27	NA	1	NA	B	NA
2017	Hanoi	3 m/F	1 dose	Negative	MT28	NA	1	NA	NA	NA
2017	Hanoi	3 m/M	1 dose	Positive	MT104	1	1	1	A	C>T
2017	Son La	2.5 m/M	0	Negative	MT27	3	1	2	NA	C
2017	Hanoi	45 d/M	0	Negative	NA	ND	ND	ND	ND	ND
2017	Hanoi	3.5 m/M	1 dose	Negative	NA	ND	ND	ND	ND	ND
2017	Hanoi	3 m/F	1 dose	Negative	NA	ND	ND	ND	ND	ND
2018	Ninh Binh	28 d/M	0	Negative	NA	ND	ND	ND	ND	ND

*MLVA, multilocus variable-number tandem repeat analysis; NA, not analyzed; ND, not determined.
†The allelic genes were analyzed only in DNA samples that yielded a complete MLVA profile.
‡*fhaB3* allele carries the SNP mutation C5330T.

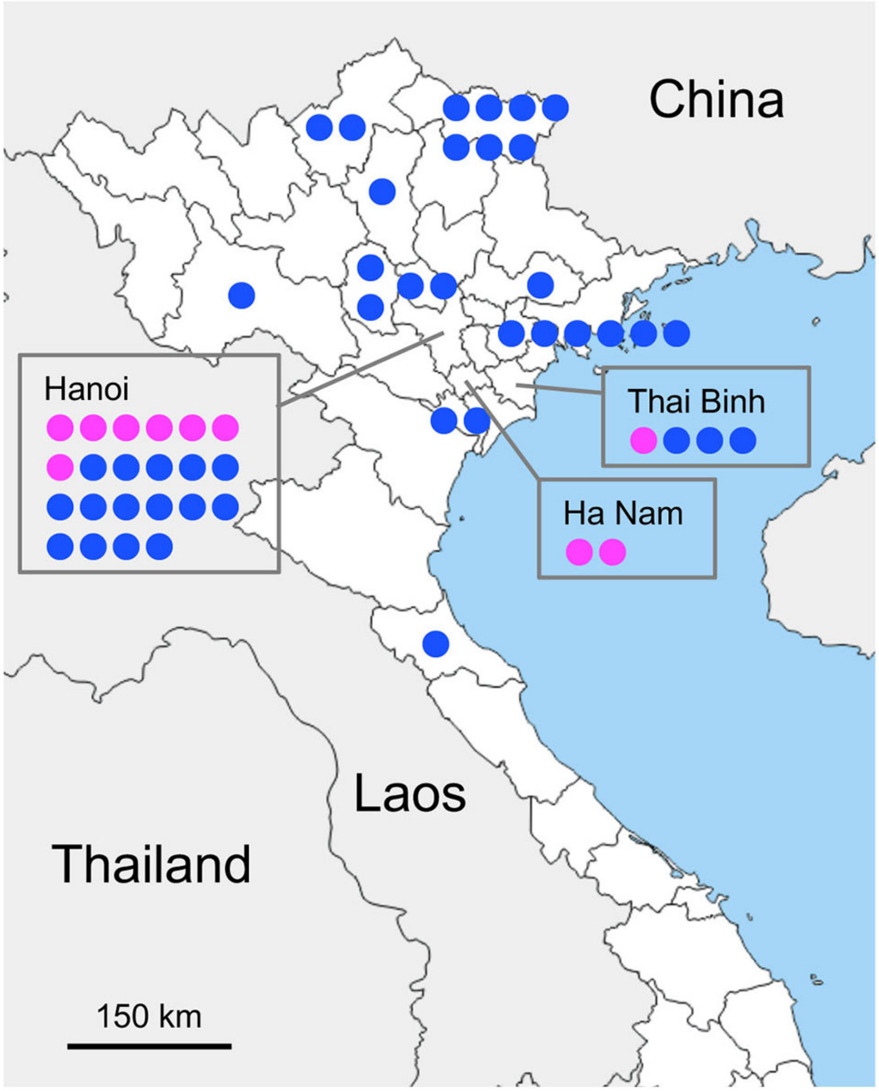

Appendix Figure 1. Geographic location of the 53 pertussis patients investigated in a study of macrolide-resistant *Bordetella pertussis*, Vietnam, 2016–2018. Red and blue circles indicate patients positive and negative for the macrolide-resistant A2047G mutation, respectively.

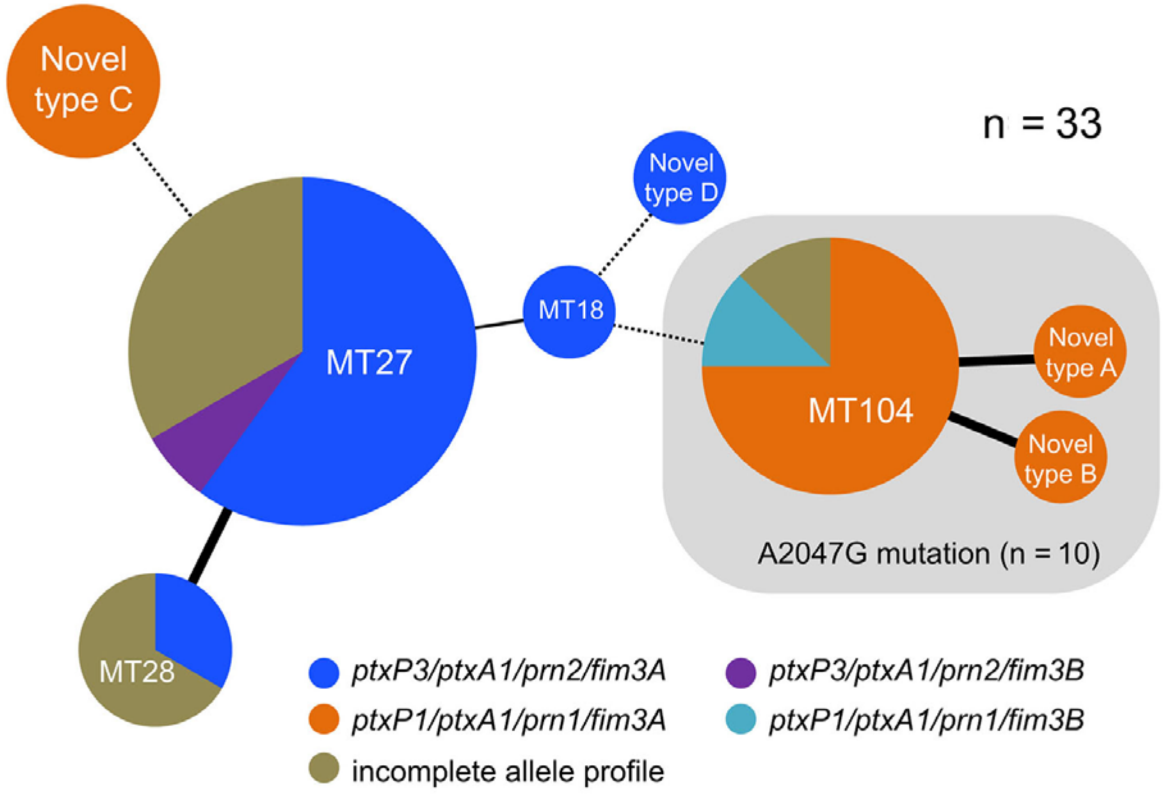

Appendix Figure 2. Minimum spanning tree revealing the genetic diversity of the *Bordetella pertussis* population, Vietnam, 2016–2017. Of the 53 DNA samples, 33 yielded a complete multilocus variable-number tandem repeat analysis profile by direct genotyping and were classified into 8 multilocus variable-number tandem repeat analysis types (MTs). The gray background represents macrolide-resistant *B. pertussis* carrying the A2047G mutation in the 23S rRNA (MT104, and novel genotypes A and B). The colors of the circles represent different allele profiles of the virulence-associated allelic genes (*ptxP/ptxA/prn/fim3*). Solid lines separate single-locus variants, dotted lines separate double-locus variants. Thick lines represent differences of one repeat at 1 variable-number tandem repeat.
